# Gene Duplication and Protein Evolution in Tick-Host Interactions

**DOI:** 10.3389/fcimb.2017.00413

**Published:** 2017-09-25

**Authors:** Ben J. Mans, Jonathan Featherston, Minique H. de Castro, Ronel Pienaar

**Affiliations:** ^1^Epidemiology, Parasites and Vectors, Agricultural Research Council-Onderstepoort Veterinary Research Onderstepoort, South Africa; ^2^Department of Veterinary Tropical Diseases, University of Pretoria Pretoria, South Africa; ^3^Department of Life and Consumer Sciences, University of South Africa Pretoria, South Africa; ^4^Agricultural Research Council-The Biotechnology Platform Onderstepoort, South Africa

**Keywords:** tick evolution, gene duplication, protein family evolution, salivary gland, blood-feeding evolution, hematophagy

## Abstract

Ticks modulate their hosts' defense responses by secreting a biopharmacopiea of hundreds to thousands of proteins and bioactive chemicals into the feeding site (tick-host interface). These molecules and their functions evolved over millions of years as ticks adapted to blood-feeding, tick lineages diverged, and host-shifts occurred. The evolution of new proteins with new functions is mainly dependent on gene duplication events. Central questions around this are the rates of gene duplication, when they occurred and how new functions evolve after gene duplication. The current review investigates these questions in the light of tick biology and considers the possibilities of ancient genome duplication, lineage specific expansion events, and the role that positive selection played in the evolution of tick protein function. It contrasts current views in tick biology regarding adaptive evolution with the more general view that neutral evolution may account for the majority of biological innovations observed in ticks.

## Evolution of hematophagy in ticks

**Figure 1:**
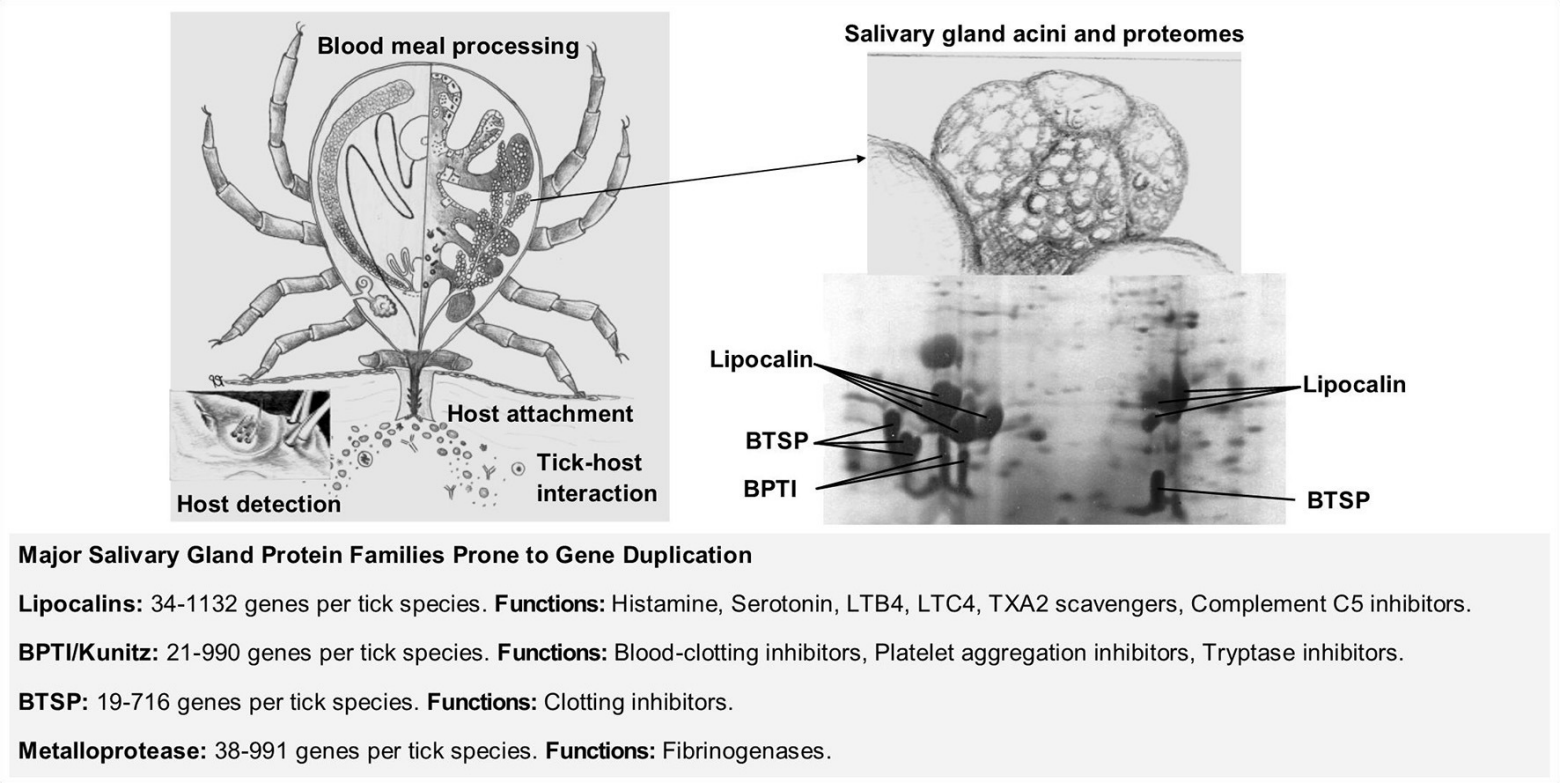
Gene duplication in the evolution of blood-feeding in ticks. The four main stages in the evolution of blood-feeding includes host detection, host attachment, tick-host interaction, and blood meal processing (Mans, 2014). Tick salivary glands play a central role in tick-host interactions by secreting the bioactive molecules that modulates host defense mechanisms. Some of these secreted proteins belong to large protein families expanded by gene duplication that can count from tens to hundreds of different genes per tick species. BPTI (Basic pancreatic trypsin inhibitor), BTSP (Basic tail secretory proteins), LTB4 (leukotriene B4), LTC4 (leukotriene C4), TXA2 (thromboxane A2). Information on protein families summarized from Mans et al. (2016).

Ticks (Ixodida) are obligate blood-feeding ecto-parasites that evolved a blood-feeding lifestyle >250 million years ago (MYA) (Mans et al., 2011, 2012, 2016). The ancestral hematophagous lineage diverged into extant families: Argasidae (soft ticks ~200 species), Ixodidae (hard ticks ~700 species), and the monotypic Nuttalliellidae (*Nuttalliella namaqua*) (Guglielmone et al., 2010). A parasitic blood-feeding lifestyle entails interaction with the vertebrate host, necessitating the evolution of mechanisms to ensure successful acquisition of a blood meal, described as the four stages of blood-feeding evolution (Figure 1), namely: host-detection, host-attachment, host-interaction, and blood meal processing (Mans, 2014). For the current study, tick-host interactions and lineage specific innovation that occurred after divergence of the main tick families are of interest. This is most apparent in the comparison of the salivary gland repertoires secreted into the host during feeding. The evolution of salivary gland protein families and protein function is mediated by gene duplication and a major issue is whether tick-host evolution is adaptive or neutral. The current review will examine these issues and aim to illuminate the general theories of gene duplication and functional evolution with tick specific examples.

## Comparative transcriptomics between hematophagous arthropods

Independent evolution of hematophagy is evident given the paraphyletic nature of blood-feeding arthropod lineages that originated at least 20 times (Ribeiro et al., 2010; Mans, 2011; Mans et al., 2016). Convergent evolution is apparent from the various solutions that blood-feeders found for their common problem, i.e., how to control the vertebrate host's defense mechanisms. A number of generalizations regarding salivary gland transcriptomes suggest that evolutionary mechanisms may be universal for all blood-feeders (Mans, 2011). As such, all blood-feeders secrete bioactive components from their salivary glands into the feeding site that target similar key host defense mechanisms. This underscores the universality of vertebrate host inflammatory and hemostatic mechanisms, since these evolved (>350 MYA), before most arthropod lineages adapted to blood feeding (Delvaeye and Conway, 2009; Conway, 2015). Unique salivary gland protein families in different arthropod lineages, suggests that salivary gland repertoires differed before adaptation to blood feeding depending on the specific lifestyle (scavenger, parasites, plant feeder, or predator) of the non-hematophagous ancestral lineage. Closely related hematophagous lineages have similar salivary gland protein families, even if they do not have similar functions and these same families are prone to gene duplication (Mans, 2011). These generalizations have predictive value for the study of uncharacterized blood-feeding lineages, where we expect targeting of key host processes, unique salivary gland repertoires and limited numbers of protein families that have been expanded by gene duplication.

## Tick salivary gland transcriptomes

Hundreds to thousands of proteins synthesized in the salivary glands of ticks target key host defense mechanisms involved in inflammation and hemostasis (Francischetti et al., 2009). Extensive tables summarizing hemostatic and immune inhibitors, host targets, protein family affiliations, and distribution of orthologs in genera and lineages were recently updated (Mans, 2016; Mans et al., 2016). Summaries of sequenced transcriptomes, secretory, and house-keeping proteins, protein family members, and largest expanded families were also tabulated (Mans, 2016; Mans et al., 2016). All tick families possess the same major salivary gland secretory protein families (Figure 1). These include lipocalins, kunitz-basic pancreatic trypsin inhibitors (BPTI), metalloprotease, serpin, trypsin inhibitor-like (TIL), cystatin, and basic tail secretory (BTSP) families (Mans et al., 2008a, 2016). However, few orthologs are shared between the different tick families and lineage specific expansions of these gene families are common (Mans et al., 2008a, 2016; Dai et al., 2012; Schwarz et al., 2014a). Of interest, is the sudden rise in described secretory family members after introduction of next-generation sequencing technologies (NGS), with an ~10-fold increase for secretory protein family members from conventional cDNA libraries (10–100s family members) to NGS (100–1,000s family members) sequenced transcriptomes. The numbers of secretory family members found in the *Ixodes scapularis* genome is closer to that observed for cDNA libraries. Whether the high levels of lineage specific expansion observed in NGS transcriptomes are real due to alternative splicing or gene duplication, or artefactual due to transcriptome assembly, taxonomic sampling, or sequence depth bias cannot be determined yet (Mans, 2016; Mans et al., 2016). However, functional analysis of the major host interacting proteins confirm that they belong to different protein families in the different tick families and therefore evolved these functions independently (Mans et al., 2002, 2016; Mans and Neitz, 2004a; Mans, 2011). The current study will discuss scenarios and evolutionary mechanisms by which these novel and different functions evolved.

## Speciation, gene duplication, and the fates of genes

Members of protein families are homologous, i.e., share a common ancestor (Koonin et al., 2002). Homology may be due to vertical descent (speciation) and such genes are orthologs or orthologous. Homology may also be due to gene duplication resulting in two or more members of the same gene within the genome and such genes are paralogs or paralogous (Fitch, 1970, 2000; Koonin, 2005; Gabaldón and Koonin, 2013). During subsequent speciation, these paralogs will become orthologs in the descendant lineages. Gene loss may occur in orthologs and paralogs, or gene duplication may occur in selective descendant lineages and can lead to phylogenetic relationships that are complex and difficult to resolve, given our dependence on information from extant lineages. Attempts to classify these relationships led to creation of designations such as co-orthologs (a gene from one species is collectively orthologous to duplicated genes in other species), inparalogs (lineage-specific gene duplications occurring after speciation), and outparalogs (lineage-specific gene duplications occurring before speciation) (Sonnhammer and Koonin, 2002; Koonin, 2005). It becomes very difficult to delineate these without a broad taxonomic sampling of closely related and divergent lineages and for most discussions, the general concepts of orthologous and paralogous genes are sufficient (Jensen, 2001). Confounding factors in ortholog identification includes domain-shuffling, acquisition/loss of new domains and alternative splicing (Gabaldón and Koonin, 2013). Most small tick proteins involved at the tick-host interface belong to single domain families (BPTI, cystatin, lipocalin, serpin, TIL), or when multi-domains are present they are generally oligomers of the same domain (e.g., BPTI, BTSP) (Francischetti et al., 2009). Even so, given the fact that extensive lineage-specific expansions occur in ticks (existence of co-orthologs and inparalogs), the identification of orthologs remains problematic.

## Orthologous genes in ticks

Orthologs generally possess similar molecular structure, function, mechanism of action, conserved residues involved in molecular interaction and domain architecture across species or lineages and can be traced to the last common ancestral lineage where this function originated (Gabaldón and Koonin, 2013). This is the basis for the universality of general cell biological processes such as transcription, translation, cellular localization, secretion, transport, metabolism, and our ability to annotate genes by homology as encapsulated in the ortholog conjecture (Gabaldón and Koonin, 2013; Rogozin et al., 2014). Since most orthologs perform vital functions in general cell biology or development, they are fixed in populations or species by negative selection. Even so, gene losses occur, orthologs acquire new or additional functions, domains are exapted for a new function (Gabaldón and Koonin, 2013). Exaptation of house-keeping functions at the tick-host interface have occurred as seen for glycolytic enolase, that also function as plasminogen activator (Díaz-Martín et al., 2013). Numerous orthologs specifically involved in tick-host interaction exist. Apyrases, biogenic amine binding proteins (BABP), enolase, metalloproteases, and defensins evolved in the last common ancestor to all ticks (Mans et al., 2016). BPTI-thrombin inhibitors, BPTI-fibrinogen receptor antagonists, and cysteinyl leukotriene scavengers evolved in the last common ancestor to soft ticks (Mans and Ribeiro, 2008a; Mans et al., 2008b). Ixodegrins, serpin-thrombin inhibitors, BPTI-thrombin inhibitors, and immunoglobulin-binding proteins evolved in the last common ancestor to hard ticks (Mans et al., 2016). Similarly, orthologs restricted to different genera exist, for example, the moubatin-clade thus far restricted to the genus *Ornithodoros* that are paralogous to the biogenic amine binding clade (Mans and Ribeiro, 2008b).

## Paralogous genes in ticks

**Figure 2:**
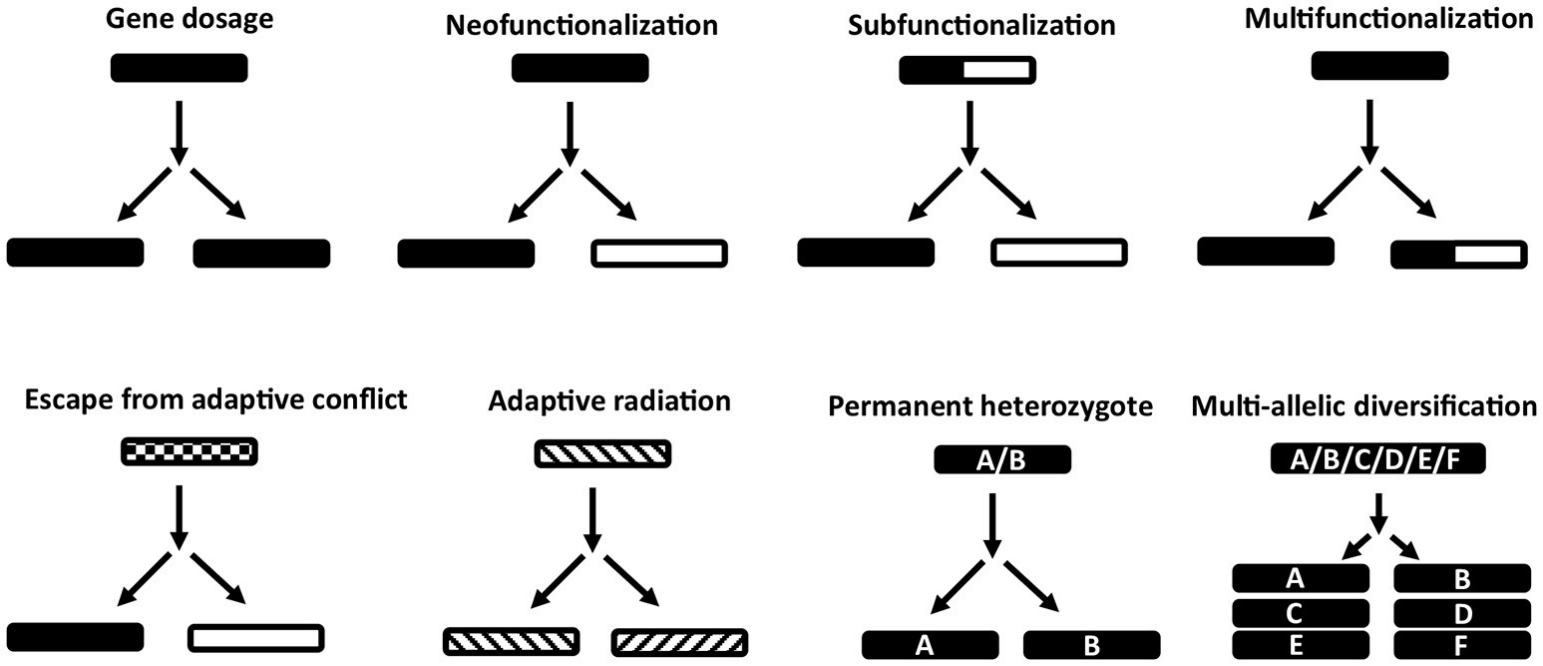
Models of gene duplication and evolution of function. Indicated are various models for the evolution of function before and after gene duplication events. Gene dosage refers to maintenance of gene duplicates with the same function. Neofunctionalization refers to the acquisition of a new function and loss of the old function in one gene duplicate. Subfunctionalization refers to the segregation of two functions in different gene duplicates that was present in the ancestral gene. Multifunctionalization refers to acquisition of a new function in one gene copy, while retaining the original function. Escape from adaptive conflict refers to an ancestral gene with functions that are overlapping or sub-functional due to exclusion effects. These functions are optimized in respective gene duplicates after duplication. Adaptive radiation refers to ancestral genes that have pre-adapted functions, which allow the evolution of similar functions in gene duplicates. Permanent heterozygote refers to heterozygotes that have better fitness than homozygotes and where gene duplication leads to fixation of both alleles in paralogs. Multi-allelic diversification refers to the case where the highest number of heterozygous individuals in a population is advantageous. Gene duplications leads to many divergent genes coding for the same function.

Once a gene duplicates several possibilities exist for its fate (Figure 2). One copy may rapidly acquire deleterious mutations and become pseudogenized (non-functionalization) (Innan and Kondrashov, 2010). Paralogs may retain the same function and if maintained by negative selection, are considered to be due to requirements of a dosage effect in house-keeping proteins, i.e., high concentrations are necessary to fulfill the requirements of the cell (Innan and Kondrashov, 2010; Rogozin et al., 2014). Genes in this category include histone and 18S, 5.8S, and 28S ribosomal RNA cassettes that occur in hundreds of copies in metazoans, including ticks (Mans et al., 2015). All copies are highly similar and maintained via concerted evolution, gene conversion or strong purifying selection (Nei and Rooney, 2005; Innan and Kondrashov, 2010). Examples of paralogs maintained for dosage effects at the tick-host interface are the savignygrins and monogrins secreted at high concentrations to target the abundant fibrinogen receptor on platelets (Mans et al., 2003a, 2008b).

Mutations may accumulate in one copy resulting in loss of the old function and evolution of a new function (neo-functionalization) and leads to the generalization that paralogs have different functions (Gabaldón and Koonin, 2013). Functions may be similar in a general sense, i.e., members of the Ran-GTP or karyopherin families all function in cellular transport and trafficking (Mans et al., 2004); tick lipocalins function as scavengers of diverse bioactive molecules such as biogenic amines (Mans et al., 2008c), cysteinyl leukotrienes (Mans and Ribeiro, 2008a), thromboxane A2 (TXA2), and leukotriene B4 (LTB4) (Mans and Ribeiro, 2008b); or BPTI inhibitors that target different serine proteases such as fXa and thrombin (Mans et al., 2002), but differ in target specificity. Functions may also differ completely, such as the platelet aggregation inhibitors that belong to the BPTI protein family, but target integrin receptors (Mans et al., 2008b).

Some genes may have multiple functions and after duplication, each gene loses one function but retains the other (sub-functionalization) (Force et al., 1999; Lynch and Force, 2000). An example in ticks may be the histamine and serotonin binding proteins, monotonin and monomine in *Argas* ticks that probably derived from a lipocalin capable of binding both histamine and serotonin in the same site (Mans et al., 2008c).

Some paralogs may also retain the original function and evolve new functions, thereby becoming multifunctional (multi-functionalization). An example in ticks would be the complement C5 inhibitors from *Ornithodoros* ticks that retained the ancestral function of scavenging LTB4, but evolved C5 complement binding activity on their βH-α2 loop (Mans and Ribeiro, 2008b).

If multiple functions are overlapping in mechanism, hindering functional optimization, gene duplication, and subsequent divergence can optimize functions; the escape from adaptive conflict model (Hughes, 1994; Des Marais and Rausher, 2008). In ticks, an example of this specific model has not yet been found, although candidates exist that would certainly benefit from this, e.g., moubatin that binds LTB4 (inhibition of neutrophil migration) and TXA2 (inhibition of platelet aggregation) in the same binding pocket at similar affinities (Mans and Ribeiro, 2008b). This increases the risk of competitive exclusion and neutralization of both functions if both antagonists were to be present in the feeding site at the same time.

Proteins with multiple functions or broad specificities may already be predisposed to evolve a new function once duplicated, the adaptive radiation model (Francino, 2005). Tick inhibitors with similar general functions may fall in this class. Positive selection has originally been promoted as the driving force for gene-level adaptive radiations, but it should be noted that neutral or even mildly deleterious mutations can also accumulate in genes and become fixed within a population through processes such as drift. It may be argued that neutral evolutionarily processes play a particularly important role for generating gene diversity and novel functions in genes with broad specificities because mutations in genes with promiscuous activity are more likely to be neutral than they are to have either a positive or negative effect on fitness.

Where heterozygous alleles have better fitness than homozygotes, gene duplication can create a permanent heterozygote (Proulx and Phillips, 2006). An extension of this model, the multi-allelic diversifying selection model, occurs when heterozygosity is advantageous at the population level (Innan and Kondrashov, 2010). In such instances, gene duplication leads to fixation of different heterozygotes under positive selection, accumulation of new alleles and gene expansion. For both these models, hyper-variable genes in ticks that possess the same function would fit in these models as proposed for genes evading the host immune system (Chmelař et al., 2016). However, even where related extant taxa have been well-sampled, in most instances it is challenging to determine which model best accounts for specific instances of family expansions because while the underlying causes for family diversification may differ the outcomes may appear similar. For example, when considering the multi-allelic diversifying model, the gene dosage model may instead be considered for gene duplicates where the active or binding site is internally located or the functional residues are restricted to a small area of the protein structure and where purifying selection only acts on these regions to conserve functionality and protein fold structure. Equally, neutral evolution and accumulation of mutations also lead to extremely diverse families that have the same or similar functions that would resemble the multi-allelic diversification model, but without the need for positive selection.

## Rates of gene duplication

Gene duplication gives rise to paralogs and is considered the major mechanism to generate genetic diversity and new functions (Lynch, 2002). The general rate of gene duplication is ~1% per gene per million years, while 50% of duplicated genes are lost every 4 million years (Lynch, 2002). In a species with ~20,000 genes (such as ticks), ~1,000 genes will become fixed over 10 million years. With regard to ticks, *I. scapularis* is the only genome available (Gulia-Nuss et al., 2016), with 2–3% predicted genomic and 7–22% transcriptomic paralogs (Van Zee et al., 2016). Paralog pairs (two duplicated genes) make up ~80% of paralogs found in the genome, indicating a single duplication event for these genes. The majority of these duplications occurred <6 MYA (Van Zee et al., 2016), which fit the expected gene duplication rate. The transcriptome data suggest a higher rate of gene duplication than expected. Genome and transcriptome differences are partly due to a higher number of genes in salivary gland transcriptomes (Valenzuela et al., 2002; Ribeiro et al., 2006), not present in the genome assembly, which only extracted gene annotations from ~57% of the genome (Gulia-Nuss et al., 2016). Conversely, other measures of completeness such as the core eukaryotic genes mapping approach (CEGMA, 248 conserved genes) and benchmarking universal single-copy orthologs (BUSCO, 2,675 conserved genes), indicate ~80 and ~69% genes represented in the *I. scapularis* genome, respectively (Hoy et al., 2016). A lack of gene coverage alone cannot account for these differences.

The only related tick species with extensive genetic data is *I. ricinus* for which salivary, midgut, and hemocyte transcriptomes were sequenced and a draft genome assembled (Chmelař et al., 2008; Schwarz et al., 2013, 2014b; Cramaro et al., 2015, 2017; Kotsyfakis et al., 2015a,b; Perner et al., 2016). Molecular clock analysis based on the mitochondrial proteins, suggests that *I. scapularis* and *I. ricinus* diverged ~9 MYA, suggesting the majority of the gene duplications detected by Van Zee et al. (2016) occurred after their divergence. It is therefore of interest that ticks exhibit extensive duplicated gene families in their salivary gland transcriptomes which seem to be lineage or species specific expansions, i.e., phylogenetic analysis indicate protein family clades that effectively consist of genes from only one species (Mans et al., 2008a, 2016; Schwarz et al., 2013). Reciprocal best hit analysis of non-redundant datasets (clustered at 95% protein identity to remove possible alleles, resulting in 20,869 *I. scapularis* and 30,641 *I. ricinus* proteins), indicates that 10,105 best hits (orthologs) occur between *I. scapularis* and *I. ricinus* (<E^−10^) (Mans et al., 2016). This corresponds with ~10,000 orthologs found between *I. scapularis* and other parasitiform mites (Dong et al., 2017). Unique proteins from each dataset with significant hits (<E^−10^) in their respective orthologous sets, comprised 23% (4,738 proteins) and 53% (16,000 proteins), respectively for *I. scapularis* and *I. ricinus*, indicating that these paralogs duplicated after speciation, or were lost in different species. In the case of *I. ricinus*, it indicates a larger gene duplication rate than *I. scapularis*. This could be due to genes missing from the *I. scapularis* assembly, true duplications in *I. ricinus* or artifacts from *de novo* assembled transcriptomes derived from NGS (Mans et al., 2016). The genome size of *I. ricinus* (~2.71 Gbp) is larger than *I. scapularis* (~2.2 Gbp) (Geraci et al., 2007; Cramaro et al., 2017), and may explain some of the paralog differences. However, for both species the number and rate of gene duplications are higher than expected, differing by ~5–16-fold from the expected number of gene duplications. With regard to major secretory protein families, the Kunitz-BPTI, BTSP, and lipocalin protein family members of *I. scapularis* possess 69 (39%, 176 total), 49 (40%, 123 total), and 47 (39%, 119 total) unique genes (duplicated per species), respectively. For *I. ricinus* there are 846 (80%, 1,058 total), 682 (82%, 834 total), and 1,011 (79%, 1,283 total) unique genes for the Kunitz-BPTI, BTSP, and lipocalin family members, respectively (Mans et al., 2016). This ranges from 40 to 80% of the total members for each family and is higher than the overall percentage of duplicated genes (7–22%) observed in the genome of *I. scapularis* (Van Zee et al., 2016). This differs by orders of magnitude from the average rate of gene duplication observed in other arthropods (Gulia-Nuss et al., 2016; Hoy et al., 2016; Dong et al., 2017), and suggests elevated gene duplication rates in ticks and most especially for secretory proteins. The question raised, is whether this number of gene duplications could have occurred since speciation, with the implication that these duplicates are an evolutionary response of the tick to a blood-feeding environment.

## Molecular arms races

The diversity in gene family members may be an adaptive response to escape the host immune system described as an escalating host-parasite arms race that leads to innovation from either side to counter-act new functions (Mans, 2011; Chmelař et al., 2016). This may take the form of new functions evolving, making defense systems on both sides more redundant (i.e., the Red Queen hypothesis). It could take the form of expansion of the same protein family to yield antigenic variants that retain the same function. These variants, secreted concurrently at low levels during feeding, evade the immune system, while still achieving a concentration necessary for immune system inhibition: the varying epitope hypothesis (Couvreur et al., 2008; Chmelař et al., 2016). The varying epitope hypothesis is a variant of the multi-allelic diversifying selection model (Innan and Kondrashov, 2010). It may also take the form of an expanded family that escapes the host's immune system by differential expression during feeding: the antigenic shift during feeding hypothesis (Chmelař et al., 2016). Supporting evidence for this may be heightened non-synonymous substitution rates (Kn/Ks > 1, positive selection), indicative of adaptive selection (Kotsyfakis et al., 2015b; Ribeiro et al., 2017).

Paralogous proteins from the same clade with similar functions such as BABPs, LTB4 scavengers or GPIIbIIIa inhibitors have appreciable sequence similarity, excluding antigenic variation to escape the immune system of the host (Mans et al., 2003a, 2008b,c; Mans and Ribeiro, 2008b). These paralogs probably fulfill a dosage requirement, since functions like scavenging of bioactive molecules or targeting abundant platelet receptors, require high concentrations of inhibitors. The only study that characterized an extensive multigene family that shows the same function, is the Salp20 group of complement inhibitors that disrupts the active C3 convertase (C3bBbP) complex by binding properdin (Valenzuela et al., 2000; Daix et al., 2007; Tyson et al., 2007, 2008; Couvreur et al., 2008). Different Salp20 genes are expressed in different individuals across the time course of feeding (Couvreur et al., 2008). This family would represent a mixed model of antigenic shift during feeding and varying epitopes hypotheses. This family has a number of gene members in both *I. scapularis* and *I. ricinus* and shows overall sequence identity of 35–75%. The members of this family are all highly N- and O-linked glycosylated, while thrombospondin repeats of properdin specifically bind sulfated glycoconjugates and glycosaminoglycans (Couvreur et al., 2008; Tyson et al., 2008). The mechanism of action among the various gene duplicates may in certain instances be sequence independent (i.e., glycosylation dependent), relaxing purifying selective constraints on the protein sequences, while retaining their function. This family would then be a very specific case of functional conservation due to post-translational modification, and not necessarily an example of antigenic shift or varying epitopes and may not be good support in favor of these hypotheses. Similarly, scavengers of bioactive molecules such as the lipocalins, may show extensive sequence variation, since their conserved binding sites are internally located and antibody responses would not necessarily block activity. Their sequence diversity may therefore not be due to antigenic variation, but may be due to genetic drift and relaxation of purifying selection.

Co-evolution of tick and host in an escalating arms race was proposed to explain the diversity of tick salivary gland proteins (Mans, 2011; Chmelař et al., 2016). In this, positive selection of secretory proteins plays an important role as a means to evade the host's immune system (Kotsyfakis et al., 2015b; Ribeiro et al., 2017). The Red Queen hypothesis was also invoked to explain gene duplication and protein evolution in salivary glands as part of an escalating arms race (Schwarz et al., 2014a). The Red Queen hypothesis is an extension of co-evolution that specifically indicates that as ticks evolve numerous functions, the host should respond in kind. Whereas, extensive gene duplication and genetic diversity is observed for ticks, the same cannot be said for the host hemostatic or even immune systems. The basic hemostatic and immune functions of the host were evolved before adaptation of ticks to a blood-feeding lifestyle and are generally conserved in mammals (Delvaeye and Conway, 2009; Conway, 2015). The Red Queen hypothesis cannot therefore be strictly applied to tick-host interactions.

## Positive selection in the face of neutral evolution and genetic drift

The current dominant view in evolutionary biology is that neutral evolution and genetic drift may have been responsible for many evolved functions and that neutral evolution should be considered as the null hypothesis that must first be rejected before adaptive explanations are considered (Lynch, 2007; Nielsen, 2009; Koonin, 2016). Positive selection, where the rate of non-synonymous substitutions in orthologs (i.e., different species) is greater than synonymous substitutions (Kn/Ks > 1), is the gold standard measure of adaptive signal (Yang and Bielawski, 2000; Nielsen, 2005; Kondrashov, 2012). When comparing closely related taxa, the majority of genes found in eukaryotic genomes have a Kn/Ks ratio ≤1, which is indicative of neutral evolution or negative/purifying selection. Overall, few genes have been identified that exhibit signatures of positive selection (Yang and Bielawski, 2000; Kondrashov, 2012). In ticks, positive selection has been detected in many secretory proteins supporting the hypothesis that ticks have adapted to a blood-feeding environment (Daix et al., 2007; Couvreur et al., 2008; Dai et al., 2012; Kotsyfakis et al., 2015b; Van Zee et al., 2016; Ribeiro et al., 2017). All of these studies calculated Kn/Ks ratios between paralogous proteins within single species or used mapping and variant detection of NGS data, where higher Kn/Ks ratios may reflect non-specific mapping of reads to paralogs. These approaches are similar to measures of volatility and liable to suffer from the same drawbacks from which no conclusion can be made regarding positive selection (Nielsen and Hubisz, 2005). In contrast, if orthologs and paralogs between *I. ricinus* and *I. scapularis* from the Salp20 families are compared, ortholog Kn/Ks ratios are below 1 (Daix et al., 2007). Detection of positive selection is complicated by the presence of paralogs in a dataset (Brieuc and Naish, 2011), and increased Kn/Ks ratios may be expected between paralogs with different functions, since their divergence entails non-synonymous substitutions. Relaxation of purifying selection in recently duplicated genes (i.e., increased rates of mutation) is also observed, but is not necessarily due to positive selection (Kondrashov et al., 2002). In ticks, the average Kn/Ks obtained for secreted proteins ranges from Kn/Ks of 1.1–1.3 (Kotsyfakis et al., 2015b; Ribeiro et al., 2017), which may be interpreted as slightly above neutral signal. However, this may be due to relaxation of purifying selection rather than positive selection, with the caveat that differences between paralogs may still be more extensive than between orthologs. An alternative definition of neutral evolution, where mutations that do not alter gene function appreciably are considered neutral, Kn/Ks-values slightly above or below 1 are still considered neutral (Nei, 2005). As yet, we cannot conclude that positive selection is evident in secretory proteins at genome or transcriptome scale for ticks until the Kn/Ks ratios for orthologs between closely related species such as *I. scapularis* and *I. ricinus* have been determined. Therefore, the null hypotheses of neutral evolution cannot be rejected in favor of an adaptive interpretation for tick-host interactions.

## Neutral evolution and genetic drift as drivers of blood-feeding evolution

If neutral evolution and genetic drift are major causes of diversity in ticks and adaptive selection is limited, the questions remain how ticks evolved the impressive array of functions exhibited at the feeding site and why elevated levels of gene duplication are observed in salivary gland families (Mans et al., 2016). One possibility lies in the opportunity that salivary gland expression offers for the evolution of new functions (Mans, 2011, 2016). Proteins destined for secretion need to maintain signals for secretion and sorting, i.e., signal peptides and sorting signals for secretory granules (Nielsen et al., 1997; Gomez-Navarro and Miller, 2016). Once a protein enters the secretory pathway, deleterious effects on cellular activities or its own function will be negated by containment within cellular and extracellular compartments, such as the secretory granule where it is packaged with other secretory proteins before being secreted into the external environment of the feeding site. Since the energetic cost of maintaining gene duplicates is relatively inexpensive (Lynch and Marinov, 2015), and deleterious mutations will not affect other cellular processes, ticks can maintain such sub-functional or even non-functional genes until they are fixed by neutral evolution and genetic drift. Neutral mutations may accumulate in these secreted sub-functional (or even non-functional genes), and be fixed as long as the secretory signals or structural determinants are maintained (Lynch et al., 2016). Once a mutation arises that is beneficial, followed by subsequent mutations that enhances this new functionality, this function may be maintained by purifying selection. This is the general case of neofunctionalization, where new functions evolve due to random neutral mutations (Innan and Kondrashov, 2010). Evolution of new function may even be more subtle, when redundant pathways or processes are targeted, starting with maintenance of the original function in the new gene duplicate until a mutation arises that changes its functional specificity slightly and allows binding to related targets with related functions. Subsequent optimization of function will lead to loss of the original function and gain of a new function. This will be a combination of the multi-functionalization, neofunctionalization, subfunctionalization, escape from adaptive conflict, and adaptive radiation models. Another possibility is for genes with broad target specificities that duplicate and subfunctionalize due to loss-of-function mutations, which result in maintenance of gene duplicates with different functions (Lynch and Force, 2000). These scenarios may explain why so many members of the same protein family target similar members in a host family (BPTI
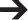
serine proteases, serpins
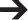
serine proteases, cystatins
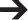
cysteine proteinases, evasins
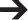
chemokines). It also implies that many proteins in salivary transcriptomes may have sub-optimal or no functions, but are not pseudogenes in the classical sense (Mudge and Harrow, 2016), since they are still transcribed, expressed and secreted. In this regard, pseudogenes may compose a large part of multigene families in genomes ranging from 25 to 60% (Nei and Rooney, 2005). It may also explain in part the lineage specific expansions observed in salivary gland protein families, since duplicated genes may rarely be lost as observed for house-keeping duplicates. The birth and growth of multigene families approximate power law behavior (Koonin et al., 2002; Koonin, 2011), with those families that start to expand due to gene duplication, accumulating more gene duplications over time. In this scenario, multigene families can rapidly expand without recourse to adaptive selection. Instead of an adaptive arms race with the host, on the battlefield of the tick-feeding interface (Chmelař et al., 2016), the salivary glands and their secretory proteins become a vast experimental playground, where the building blocks can be tested and changed by neutral evolution without being discarded. The latter scenario posits the null hypothesis of neutral evolution in terms of tick-host interactions and the general argument may hold for blood-feeding arthropods in general, since all show expansion of lineage specific protein families (Mans, 2011).

## Dating of multigene families

Gene duplication linked with molecular sequences and phylogenetic analysis offers the promise of dating gene duplication events (Kumar, 2005). This could allow determination of gene duplication rates and estimation of the emergence time of new functions. However, without accurate constraints on nodes, the high level of divergence in salivary gland paralogs could lead to over-estimation of divergence times using molecular clock models. While this is an acknowledged problem in molecular clock dating techniques (Kumar, 2005), scenarios arise where divergence estimations for *I. ricinus* and *I. scapularis* based on analysis of the BPTI family indicated multiple divergence dates for these species in different clades of the same protein family (Schwarz et al., 2014a). As such, dates ranged from 10 to 66 MYA for divergence of *I. ricinus* and *I. scapularis*, and from 8 to 60 MYA for clades that were clearly lineage specific expansions in *I. ricinus* (Schwarz et al., 2014a). This implies non-clock-like behavior in different clades (Mans et al., 2016). Since *I. scapularis* and *I. ricinus* are genetically closely related and estimated to have diverged ~9 MYA, only one divergence date is possible for these species, while lineage specific expansion likely occurred <6 MYA in both species (Van Zee et al., 2016). This negates most of the evolutionary conclusions made in the Schwarz et al. (2014a) study. It was suggested that clade G6 is fundamental in understanding evolution of the BPTI inhibitors in *I. ricinus* (Schwarz et al., 2014a). This clade is the largest monophyletic clade (lineage specific expansion) and the fastest evolving clade (diverging around 70 MYA as estimated with the molecular clock). However, as indicated, the divergence observed could only have occurred <9 MYA and therefore represents an interesting view on the gene duplication rate in this family. It was suggested that accelerated evolution is evident from this analysis based on the number of members (Schwarz et al., 2014a). However, counts of the numbers of orthologs (15), paralogs (150) and gene duplications per orthologous clade (Average = 4.9, Range = 1–22), indicate that any given orthologous clade did not expand excessively (≤25 duplications/paralogous clade in 5 million years). Given power law behavior this number of gene duplications may be well within the expected norm for the major secretory protein families. If it is taken into account that some genes may be pseudogenes (Nei and Rooney, 2005), that some orthologous groups may be underrepresented in the *I. scapularis* genome, or that the *I. ricinus* transcriptome may be artefactually inflated, then the proposed accelerated evolution is not obvious. What this section highlights is the need for robust and careful analysis when multigene families are analyzed, specifically with regard to divergence time estimates. Efforts to establish which genes are truly expressed as proteins and functional at the feeding site would also assist in making phylogenetic analysis more robust. Taxonomic sampling of closely related lineages to assist in the identification of orthologous clades to prevent artificial interpretations of species specific expansions is necessary and important to correlate divergence dates for clades expected to have diverged at the time of speciation. Conversely, inclusion of highly divergent sequences from distantly related species does not increase nodal confidence, since small families such as the BPTI fold are likely to have accumulated saturated substitutions. In these cases phylogenetic trees may be obtained that given the low nodal support are basically non-informative (Dai et al., 2012). Given the expansion of tick sequences, future analyses of multigene families may need to focus exclusively on specific lineages or genera, with the emphasis that adequate taxon sampling in these lineages or genera is necessary to delineate orthologous relationships and lineage specific expansions. Inclusion of soft and hard tick sequences in the same analysis leads to the interesting conclusion that argasid and prostriate ticks diverged 155 or 100 MYA, while metastriate ticks diverged 193 MYA (Schwarz et al., 2014a), or that hard and soft tick sequences are interspersed throughout the tree with low nodal support and no evidence for orthology (Dai et al., 2012). This may be expected when divergent paralogous genes are analyzed, which beyond sharing a common ancestor in the ancestral tick lineage, may be separated by multiple gene duplication events. Analysis of multigene families should therefore be performed within a biological context, taking into account recognized phylogenetic relationships (Null hypothesis) and where phylogenetic data deviate from this, data that is not robust should be discarded. Conversely, multigene families and paralogous clades offer the opportunity to reduce uncertainty in molecular clock estimates by using the principle that divergence of paralogous clades from different species by definition occur at the same time. Different nodes can then be constrained by cross-calibration or cross-bracing to ensure that they will have similar ages, thereby improving time estimates across the whole tree (Shih and Matzke, 2013; Zhaxybayeva et al., 2013).

## Phylogenetic analysis of functional evolution

While adaptive selection may not be a major phenomenon in the evolution of blood-feeding behavior, ancestral reconstruction of functional evolution allows for a historical description of “adaptation to a blood-feeding environment.” This approach aims to answer the how, when and where functions evolved in different tick lineages (Mans et al., 2016). Phylogenetic analysis linked with functional annotation allow for the reconstruction of the evolution of functions in paralogous genes or testing of orthologous relationships to define the origins of functions in ancestral lineages. However, most phylogenetic analyses of salivary gland protein families are used to summarize or visualize transcriptome data and not to construct explicit functional evolution scenarios. Little information regarding evolution of function is extracted from these analyses beyond confirmation of lineage specific expansion and assignment of proteins to clades (Valenzuela et al., 2002; Francischetti et al., 2005, 2008a,b, 2011; Couvreur et al., 2008; Mans et al., 2008a; Anatriello et al., 2010; Karim et al., 2011; Ribeiro et al., 2011, 2012; Garcia et al., 2014; Tan et al., 2015). This is partly due to the paucity in available functional information and, given the increase in transcriptome studies and the lag in functional studies are likely to continue into the future. However, it is also due to the extensive lineage specific expansions observed in protein families, making identification of orthologs in different genera difficult, since the families are now becoming so large that accurate multiple alignments and well-supported phylogenetic trees are not feasible goals anymore (Mans et al., 2016). On the other hand, phylogenetic analyses have also been performed to analyze protein families during their functional characterization. This was done in the BPTI family for hemostatic (Mans et al., 2002, 2003a, 2008b; Francischetti et al., 2004), ion channel (Paesen et al., 2009), and tryptase inhibitors (Valdés et al., 2013). Analysis of the BPTI family in general was performed for *I. ricinus* (Schwarz et al., 2014b; Valdés and Moal, 2014), *I. scapularis* (Dai et al., 2012), and *R. microplus* (Louw et al., 2013). For the lipocalin family it has been performed for the biogenic amine binding proteins (Mans et al., 2003b, 2008c; Mans and Neitz, 2004b; Mans and Ribeiro, 2008a,b; Díaz-Martín et al., 2011; Valdés et al., 2016), LTB4 and TXA2 binding proteins (Mans and Ribeiro, 2008b), LTC4 binding proteins (Mans and Ribeiro, 2008a; Manzano-Román et al., 2016), japanin (Preston et al., 2013), and savicalin (Cheng et al., 2010). Lipocalins have also been analyzed in general for *I. ricinus* (Beaufays et al., 2008; Konnai et al., 2011), *Ornithodoros savignyi* (Mans et al., 2003b; Mans and Neitz, 2004b), and *R. microplus* (Rodriguez-Valle et al., 2013). Evolution of the serpin family has been studied in *A. americanum* (Mulenga et al., 2007; Porter et al., 2015), *I. scapularis* (Mulenga et al., 2009), and *R. microplus* (Tirloni et al., 2014). Cystatins were analyzed in *I. scapularis* (Kotsyfakis et al., 2006; Ibelli et al., 2013) and novel families described using phylogenetic analysis (Mulenga et al., 2013). It is clear that phylogenetic analysis has become a central part of salivary gland protein family characterization. However, the majority of these studies still use phylogenetic analysis to place their proteins of interest within a context of other proteins with known function, predict function by homology, or attempt to describe domain evolution in general. Reconstruction of functional evolution of paralogous clades is still limited and mainly due to the lack of knowledge regarding the functions and functional mechanisms of the majority of secretory proteins.

Studies that addressed scenarios for the evolution of function between paralogs, include the clotting and platelet aggregation inhibitors from soft ticks (Mans et al., 2002, 2008b), evolution of biogenic amine binding (Mans et al., 2008c) and evolution of LTB4, TXA2, and complement C5 inhibitors in the moubatin clade of soft tick inhibitors (Mans and Ribeiro, 2008a). For BPTI proteins it has been proposed that soft tick thrombin, fXa and fibrinogen receptor inhibitors share a common evolutionary pathway (Mans et al., 2002, 2008b). Both thrombin and platelet aggregation inhibitors have orthologs in the *Argas* and *Ornithodoros* genera, implying that blood-clotting and platelet aggregation inhibitors evolved in the last common ancestor to soft ticks, which was dated at ~234 ± 25 MYA using mitochondrial protein analysis (Mans et al., 2008b, 2012).

**Figure 3:**
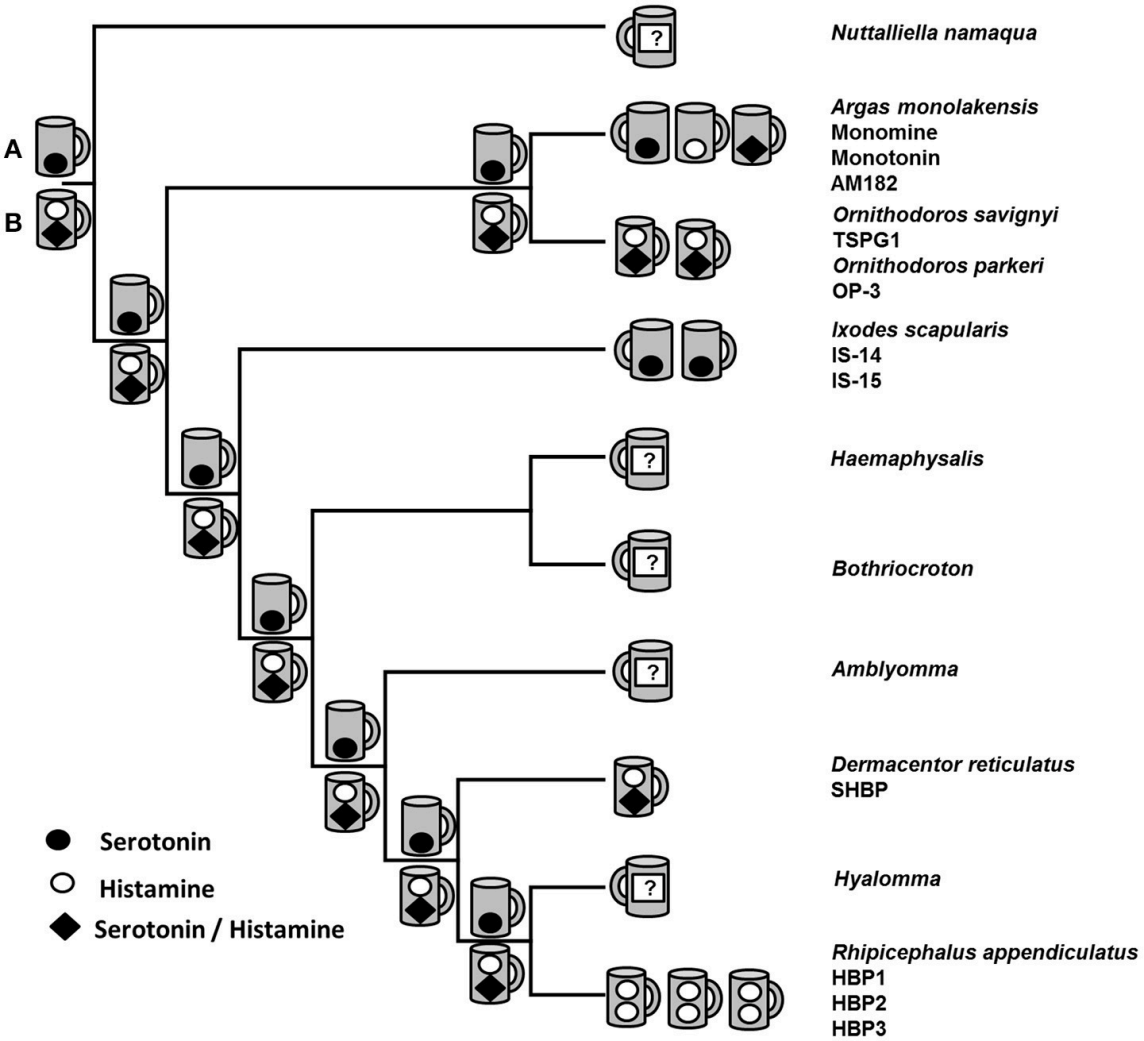
Evolutionary scenarios in the reconstruction of biogenic amine binding function. Indicated are the phylogenetic relationships of various tick genera as determined with mitochondrial analysis (Mans et al., 2012). Terminal nodes represent extant lipocalins for which empirical histamine or serotonin binding data exist. The number of lipocalins for which experimental data have been generated are indicated as cups (lipocalin~calyx~cup) that bind histamine in the upper binding site and serotonin and/or histamine in the lower binding pocket. Names of characterized lipocalins are indicated below their genus names. Internal nodes represent two possible ancestral binding site hypotheses: **(A)** The ancestral BABP had a single lower serotonin-binding site, or **(B)** the ancestral BABP had an upper histamine and lower histamine/serotonin-binding site.

The biogenic amine binding clade represents an interesting model for functional evolution since members exist that can bind histamine in an upper binding pocket (or not) and histamine and/or serotonin in a lower binding pocket (Paesen et al., 1999, 2000; Sangamnatdej et al., 2002; Mans et al., 2008c). Several models for evolution of biogenic amine binding therefore exist that cannot yet be adequately resolved (Figure 3). Parsimony analysis of the BABPs suggested that the ancestral biogenic amine bound in the lower binding pocket of the lipocalin barrel was serotonin and that histamine binding originated several times independently, both in the lower and upper binding sites (Mans et al., 2008c). This would represent the neofunctionalization model of biogenic amine binding. Alternatively, the ancestral BABP possessed both an upper histamine-binding and a lower histamine/serotonin-binding site as retained in lipocalins from *Ornithodoros* and *Dermacentor* (Sangamnatdej et al., 2002; Mans et al., 2008c). Subsequent gene duplication and subfunctionalization (or escape from adaptive conflict, since histamine and serotonin shares the same lower binding pocket) led to loss of the upper binding site and lipocalins that either binds histamine or serotonin in the lower binding site as observed for monotonin and monomine from *Argas*, or the *Ixodes* serotonin binding lipocalins (Mans et al., 2008c). In the case of *I. scapularis*, the histamine-binding gene may have been lost after subfunctionalization, although a much larger screening of genes is necessary to confirm this. A similar scenario exists for the histamine-binding lipocalins from *Rhipicephalus appendiculatus* that seem to bind only histamine (Paesen et al., 1999). These alternative scenarios may be resolved once more members are empirically verified from different genera, emphasizing the need for a broad taxon sampling in evolutionary studies. The evolutionary models presented here aim to distill our current knowledge on BABP evolution from experimentally verified data. It should be noted that the evolutionary pathways may be more complex, since an expansion of proteins with the biogenic amine binding motif is observed in all tick transcriptomes, leading to potentially tens to hundreds of biogenic amine binders in each species. It is likely that all different models of functional evolution after gene duplication are present in this family, making it a particularly interesting study model.

Another clade with detailed functional analysis that allows for dissection of evolutionary scenarios are the moubatin-clade of LTB4, TXA2 and complement C5 inhibitors (Mans and Ribeiro, 2008a). This clade shares a sister relationship with the BABP clade and is found only in the Ornithodorinae. It therefore represents paralogous gene duplication that occurred in the last common ancestor of the Ornithodorinae. The residues conserved for TXA2 and LTB4 binding are conserved across the family, suggesting that this is the ancestral function of this clade. Interestingly enough, this is a potential case for escape from adaptive conflict. In this regard, glycine 85 located inside the lipocalin beta-barrel is important for TXA2 binding, since other more bulky residues such as arginine interferes sterically with binding. TSGP2 that possesses an arginine at this position is unable to bind TXA2, but can be rescued by a R85G mutation. The same R85G mutation occurs in *Ornithodoros moubata* complement inhibitor (OMCI) and would suggest that LTB4 binding has been optimized in these two members of the clade. Both of these lipocalins (and TSGP3) target complement C5 using a very specific βH-α2 loop (Mans et al., 2008a; Jore et al., 2016). This would suggest multi-functionalization and escape from adaptive conflict by specialization of LTB4 and TXA2 binding. Recent research suggests that targeting of the complement cascade, neutrophils and platelet aggregation are closely interlinked (Deppermann and Kubes, 2016). Multi-functional proteins that target related mechanisms may evolve, not because of adaptive selection, but rather because of neutral evolution resulting in redundancy ultimately leading to beneficial mutations.

## Whole genome duplication in ticks

Gene duplication may occur for individual genes in a stochastic manner across the genome. On the other hand, large-scale gene duplication events that include chromosome or whole genome duplications give rise to many gene duplicates at once and are an important source for the generation of gene copies and as a catalyst for adaptation to new environments and speciation (Nei, 2005; Van de Peer et al., 2009). In ticks, the extensive duplication events observed in transcriptomes were suggested to be due to possible genome duplications in hard ticks (Ribeiro et al., 2006; Dai et al., 2012). This may be partly supported by the increase in chromosome numbers observed in ticks when compared to other parasitiform mites, with mesostigmatid mites having 2- to 3-fold fewer chromosomes (2*n* = 8 ± 0.3), compared to soft (2*n* = 22 ± 4) and hard (2*n* = 22 ± 2) ticks (Oliver, 1977; Mans and Neitz, 2004a). Analysis of the *I. scapularis* genome found no evidence for a genome duplication event (Van Zee et al., 2016). However, the analysis focused on recent gene duplication events dated <6 MYA. Given the similarities in chromosome numbers of hard and soft ticks, genome duplication in ticks may have occurred in the ancestral tick lineage (>290 MYA) and given that the majority of duplicated genes go extinct, may not be easily detectable since detection of whole genome duplication becomes increasingly difficult with age (Vanneste et al., 2013). The major contributor to genome size in ticks are repetitive elements with 66% contributing to the genome size of *I. scapularis* and 69% for *R. microplus* (Ullmann et al., 2005). It is not yet clear what proportion of the expanded genome size of *I. ricinus* may be attributed to repetitive elements (Cramaro et al., 2017), but it is likely that most of the genome size differences observed between tick species are not due to gene duplications, but rather the universal abundance of repetitive elements and non-coding DNA (Mans et al., 2016). Larger genome sizes of ticks compared to other mites is not evidence of genome duplication *per se* (Geraci et al., 2007). We may tentatively propose that chromosome numbers suggest a whole genome duplication in the ancestral lineage of ticks and this may have been associated with adaptation to blood-feeding. Subsequent divergence and stochastic gene duplication may explain more recent differences observed in tick transcriptomes.

## Conclusions

Gene duplication remains a major factor in the evolution of blood-feeding behavior of ticks, even if most new functions and the evolution of secretory families in ticks may undergo neutral evolution. Hypotheses on adaptive evolution should first reject the null hypothesis that evolution did not occur via neutral evolution. More rigorous analysis is also necessary to characterize paralogous gene families, especially where molecular dating is concerned. Analysis of the literature indicates that our understanding of functional evolution is still limited, since few studies aim to reconstruct evolutionary pathways. Even so, the increase in taxon sampling of salivary gland transcriptomes holds the promise that we will be able to get an accurate estimate of the number of orthologous clades distributed across various tick lineages and the number of functions encoded in the tick sialoverse.

## Author contributions

BM, JF, MdC, and RP conceptualized the manuscript. BM and JF wrote the manuscript. BM and RP prepared the figures. BM, JF, MdC, and RP revised and edited the manuscript.

### Conflict of interest statement

The authors declare that the research was conducted in the absence of any commercial or financial relationships that could be construed as a potential conflict of interest.
